# Efficacy and safety of artemisinin-based combination therapy and the implications of *Pfkelch13* and *Pfcoronin* molecular markers in treatment failure in Senegal

**DOI:** 10.1038/s41598-020-65553-5

**Published:** 2020-06-01

**Authors:** Mamadou Alpha Diallo, Mamadou Samb Yade, Yaye Die Ndiaye, Ibrahima Diallo, Khadim Diongue, Saidou Abdoul Sy, Mouhamad Sy, Mame Cheikh Seck, Mouhamadou Ndiaye, Baba Dieye, Jules François Gomis, Djiby Sow, Awa Bineta Dème, Aida Sadikh Badiane, Daouda Ndiaye

**Affiliations:** 1 Department of Parasitology and Mycology, Cheikh Anta Diop University, Avenue Cheikh Anta Diop, BP 5005 Fann, Dakar, Senegal; 2 National Malaria Control Program (NMCP), Rue Aimé Césaire, Fann Résidence, Dakar, Senegal

**Keywords:** Malaria, Parasitology

## Abstract

In 2006, Senegal adopted artemisinin-based combination therapy (ACT) as first-line treatment in the management of uncomplicated malaria. This study aimed to update the status of antimalarial efficacy more than ten years after their first introduction. This was a randomized, three-arm, open-label study to evaluate the efficacy and safety of artemether-lumefantrine (AL), artesunate-amodiaquine (ASAQ) and dihydroartemisinin-piperaquine (DP) in Senegal. Malaria suspected patients were screened, enrolled, treated, and followed for 28 days for AL and ASAQ arms or 42 days for DP arm. Clinical and parasitological responses were assessed following antimalarial treatment. Genotyping (*msp1*, *msp2* and 24 SNP-based barcode) were done to differentiate recrudescence from re-infection; in case of PCR-confirmed treatment failure, *Pfk13* propeller and *Pfcoronin* genes were sequenced. Data was entered and analyzed using the WHO Excel-based application. A total of 496 patients were enrolled. In Diourbel, PCR non-corrected/corrected adequate clinical and parasitological responses (ACPR) was 100.0% in both the AL and ASAQ arms. In Kedougou, PCR corrected ACPR values were 98.8%, 100% and 97.6% in AL, ASAQ and DP arms respectively. No *Pfk13* or *Pfcoronin* mutations associated with artemisinin resistance were found. This study showed that AL, ASAQ and DP remain efficacious and well-tolerated in the treatment of uncomplicated *P. falciparum* malaria in Senegal.

## Introduction

Since the abandoning of chloroquine, due to high rate of resistance, the Senegal national malaria control program (NMCP) adjusted its national guidelines for the treatment of uncomplicated malaria by introducing sulphadoxine-pyrimethamine (SP) in 2003, and artemisinin-based combination therapy (ACT) in 2006, complying with the World Health Organization (WHO) recommendations^1^. This policy review was adopted as ACTs were proven to be the most effective treatment in the context of resistance to chloroquine and other antimalarial drugs^2^. The scaling up of ACTs at national level has largely contributed to the reduction in morbidity and mortality linked to malaria in Senegal^3^. Artemether-lumefantrine (AL) and artesunate-amodiaquine (ASAQ) are used as first-line treatment but dihydroartemisinin-piperaquine (DP) has been widely used to compensate for antimalarial drug shortages in 2010 and 2011.

In 2008, ASAQ combination was subsidized by the government and its partners, and was made available at a very low price in public health facilities and private pharmacies^4^. Since May 2010, ACTs were offered free of charge in the public sector^5^.

However, the wide use of ACT may exert selective pressure on *Plasmodium falciparum* populations over time. Recent studies in Southeast Asia (SEA) have shown a decrease in the effectiveness of ACTs such as artesunate used as monotherapy^6,7^. Although this situation is not yet reported in Africa, it is likely that this resistance could potentially spread in Africa as history showed for chloroquine^8^. This is particularly important as a previous *ex vivo* study in Senegal showed that parasites became less sensitive to amodiaquine, artemisinin and chloroquine over time^9^.

Thus, simple method for monitoring antimalarial drugs is crucial for proper management of clinical cases and early detection of resistance. In accordance with WHO recommendations, the efficacy of first and second-line antimalarial drugs should be evaluated at least once every two years at all sentinel sites^10^.

*In vivo* drug clinical trials are the gold standard for assessing the therapeutic efficacy of antimalarials^11^. In addition, *in vitro* assays for the sensitivity of human malaria parasites to antimalarial drugs provide useful complementary data from drug-efficacy surveillance^12^.

In Senegal, the NMCP and its partners regularly conduct therapeutic efficacy study (TES) and molecular markers of antimalarial drug resistance are monitored.

Mutations in the *Pfkelch13* (*Pfk13*) propeller domain were found to be associated with delayed parasite clearance *in vitro* and *in vivo* in SEA. These mutations were discovered in the laboratory by long-term *in vitro* selection using culture-adapted *Plasmodium falciparum* isolates by stepwise increases in artemisinin exposure^13^. Although the frequency of mutant alleles strongly correlated with the resistance to artemisinin, the relevance of these mutations on artemisinin resistance in other endemic areas has been subject of debates^14,15^. Using the same methodology as above^13^, but with *P. falciparum* isolates from Senegal, a recent study found no selected mutations on the *Pfk13*; instead, genetic variants (G50E, R100K, and E107V) of the gene encoding the actin-binding protein *P. falciparum* coronin (*Pfcoronin*) were found to reduce the susceptibility of the parasite^15^. Thus, *Pfcoronin*, which is structurally similar to *Pfk13*, is believed to be a strong predictor of potential artemisinin resistance in Senegal and even probably elsewhere in Africa. However, for those mutations to be validated as a marker for artemisinin resistance there should be, at least, a correlation with delayed clearance in clinical studies.

Thus, we aimed to analyze simultaneously the sequences of *Pfk13* and *Pfcoronin* on samples that were subject to treatment failure, along with the routine TES of ACTs.

## Results

### Baseline characteristics

A total of 796 patients suspected for malaria were screened during the study period. Of these, 496 (62.3%) met the inclusion criteria and were enrolled accordingly as shown in Fig. 2.

**Figure 1:**
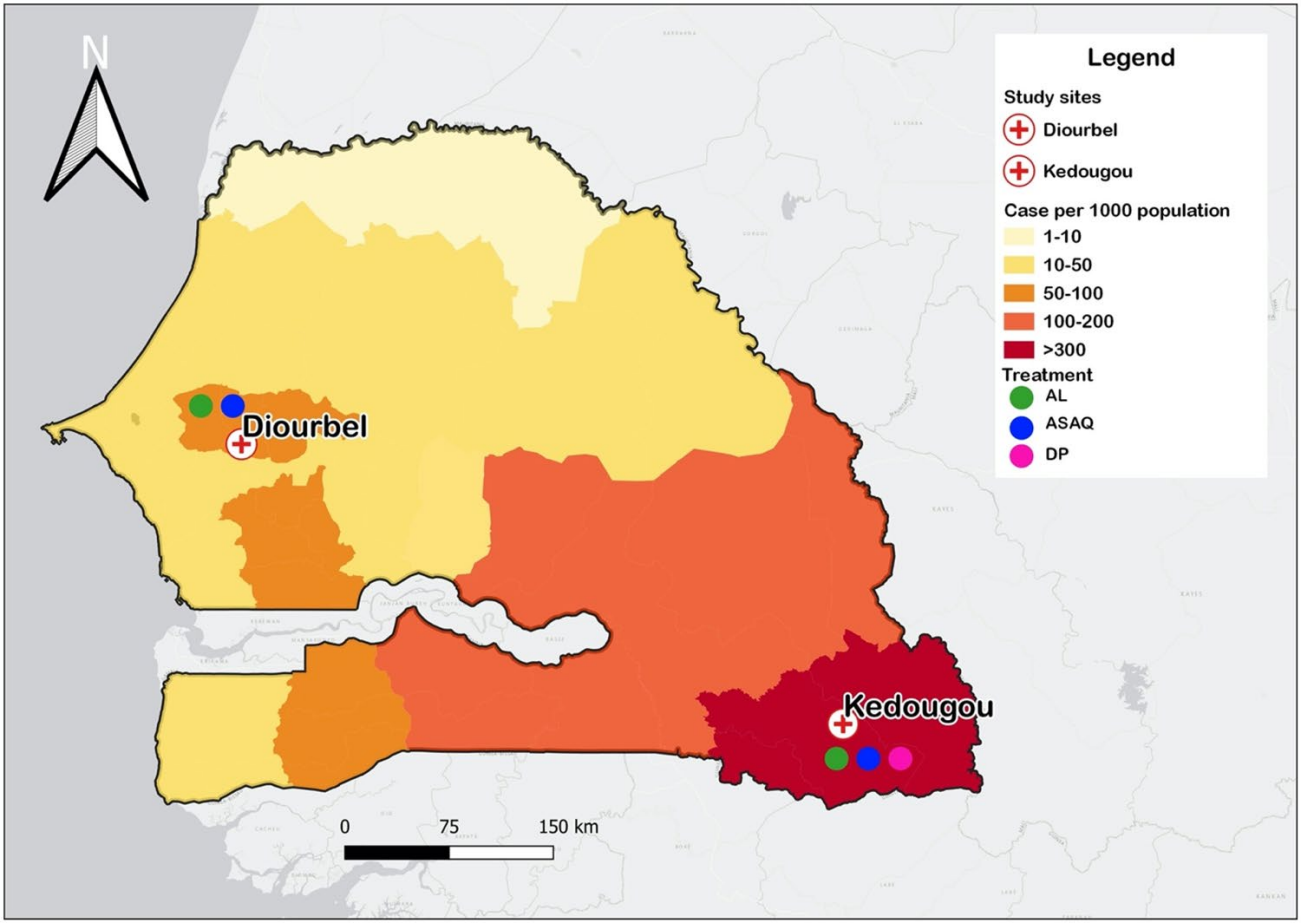
Map of Senegal showing the two NMCP sentinel sites covered in the 2018 therapeutic efficacy study (TES). This map was created using QGIS v. 3.8.3-Zanzibar.

**Figure 2:**
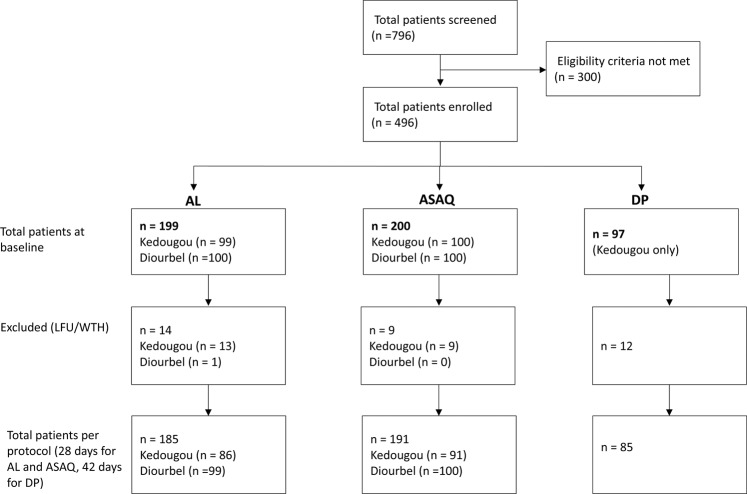
Flow chart showing screen and enrollment of study patients.

At baseline (day 0), the male to female sex ratio was largely dominated by males in Diourbel (5.1) while in Kedougou this was balanced (0.9). Thirty-six (12.2%) were under-five years old children, while this age group was not found in patients enrolled in Diourbel. Age group 5–15 years represented 117 patients (58.5%) and 163 patients (55.1%) in Diourbel and Kedougou respectively. Adult patients represented 83 (41.5%) and 97 (32.8%) in Diourbel and Kedougou respectively. Mean weight was 39.3 kg and 36.1 kg in Diourbel and Kedougou respectively. Mean body temperature was 38.3 °C and 38.1 °C in Diourbel and Kedougou respectively. Mean parasitemia was estimated at 11939 parasites/ul and 12991 parasites/ul in Diourbel and Kedougou respectively (Table 1).

**Table 1:** Baseline characteristics of the study participants.

	Diourbel	Kedougou
Number of enrolled patients	200	296
Ratio male/female	167/33	143/153
Age group (years)		
adults	83	97
5 to 15 years	117	163
under 5 years	0	36
Age (years)		
mean (sd)	17.1 (11.9)	13.3 (9.7)
range (min-max)	5–64	1–49
Weight (kg), day 0		
mean weight (sd)	39.3 (18.8)	36.1 (18.8)
range (min-max)	13–93	9–93
Temperature (°C), day 0		
mean temperature (sd)	38.3 (1)	38.1 (0.8)
range (min-max)	36.2–41.0	36.0–42.0
Parasitemia (density/µl), day 0		
mean (geometric) parasitemia	11939	12991
range (min-max)	1000–99750	1010–99828

### Clinical and parasitological outcomes

Of the 496 patients enrolled, 199, 200 and 97 were assigned to AL, ASAQ and DP arms respectively. For AL and ASAQ arms, 185 out of 199 and 191 out of 200 completed the follow-up at day-28 respectively while for the DP arm, 85 out of 97 completed the follow-up at day-42 (Fig. 2).

Table 2 presents the treatment outcome by sites and drugs based on per protocol analysis.

**Table 2:** Summary of treatment outcomes based on Per Protocol analysis in Diourbel and Kedougou, 28 days follow up of AL and ASAQ and 42 follow up of DP.

	Sites	drug	N	LFU/WTH n (%)	PD day 3 n (%)	ETF % (IC95%)	LCF\(IC95%)	LPF%(IC95%)	ACPR%(IC95%)	Recrudescence n (%)	Re-infection n (%)
PCR non-corrected	**Diourbel**	AL	100	1 (1)	0	0	0	0	100.0 (96.3–100.0)		
		ASAQ	100	1 (1)	0	0	0	0	100 (96.4–100.0)		
	**Kedougou**	AL	99	13 (13.1)	0	1.2 (0.0–6.3)	1.2 (0.0–6.3)	1.2 (0.0–6.3)	96.5 (90.1–99.3)		
		ASAQ	100	9 (9.0)	1 (1)	0	0	0	100 (96.0–100.0)		
		DP	97	11 (11.6)	0	0	0	3.6(0.7–10.1)	96.4 (89.9–99.3)		
PCR corrected	**Diourbel**	AL	100	1 (1%)	0	0	0	0	100 (96.3–100.0)		
		ASAQ	100	1 (1%)	0	0	0	0	100 (96.4–100.0)		
	**Kedougou**	AL	98	14 (14.3)	0	1.2 (0.0–6.5)	0	0	98.8 (93.5–100.0)	1 (1.2)	2 (2.4)
		ASAQ	100	9 (9.0)	1 (1)	0	0	0	100 (96.0–100.0)	0	0
		DP	97	12 (12.6)	0	0	0	2.4 (0.3–8.4)	97.6 (91.6–99.7)	2 (2.5)	1 (1.2)

Abbreviations: LFU/WTH lost to follow-up/withdrawn, PD3 positive on day 3, LCF late clinical failure, LPF late parasitological failure, ACPR adequate clinical and parasitological response, ASAQ artesunate + amodiaquine, AL artemether + lumefantrine, DP dihydroartemisinin + piperaquine.

In Diourbel, PCR non-corrected ACPR was 100.0% (95%CI: 96.3–100.3) in both AL and ASAQ arms.

In Kedougou, PCR non-corrected ACPR were 96.5% (95%CI: 90.1–99.3), 100% (95%CI: 96.0–100.0) and 96.4% (95%CI: 89.9–99.3) in AL, ASAQ and DP arms respectively; when PCR-corrected, ACPR became 98.8% (95%CI: 93.5–100.0), 100% (95%CI: 96.0–100.0) and 97.6% (95%CI: 91.6–99.7) respectively.

Treatment failures (TF) were noted in Kedougou, as following: a) one ETF at day 3 in the ASAQ arm; b) three TF in the AL arm: 1 LCF at day 7 and 1 LPF at day 21, giving PCR-uncorrected failure rate of 3.5% (95% CI: 0.0–6.3%); c) three TF in the DP arm: one LPF at day 14, one LPF at day 35, and one LPF at day 42. After PCR correction, one sample in the AL arm and two in the DP arm (at day 14 and 35) were confirmed to be recrudescence cases.

Considering the 10% of AL and ASAQ patients followed up to 42 days, there was three LPF in the AL arm and when PCR-corrected this yielded to one confirmed recrudescence (day 35) as shown in Table 3.

**Table 3:** Summary of treatment outcomes based on Per Protocol analysis of the 10% extra patients of AL and ASAQ arms followed up to 42 days.

	Sites	drug	N	LFU/WTH	PD day 3 n	ETF n	LCF n	LPF n	ACPR n/n	Pf recrudescence n	Pf re-infection n
PCR uncorrected	**Diourbel**	AL	10	0	0	0	0	0	10/10		
		ASAQ	10	2	0	0	0	0	8/8		
	**Kedougou**	AL	10	2	0	1	0	3	4/5		
		ASAQ	10	1	0	0	0	0	9/9		
PCR corrected	**Diourbel**	AL	10	0	0	0	0	0	10/10		
		ASAQ	10	2	0	0	0	0	8/8		
	**Kedougou**	AL	10	4	0	1	0	1	4/5	1	2
		ASAQ	10	1	0	0	0	0	9/9		

Abbreviations: LFU/WTH lost to follow-up/withdrawal, PD3 positive on day 3, LCF late clinical failure, LPF late parasitological failure, ACPR adequate clinical and parasitological response, ASAQ artesunate + amodiaquine, AL artemether + lumefantrine, DP dihydroartemisinin + piperaquine.

### Genotyping and molecular markers of drug resistance (*Pfk13* and *Pfcoronin*)

*msp1* and *msp2* genotyping and 24 SNP-based barcoding results were in agreement on all tested samples. Among the nine treatment failures, one was classified as ETF (recurrence at day 2), six samples were successfully genotyped and two samples gave negative PCR results on the day of recurrence. Thus, the successfully genotyped samples showed three cases of recrudescence and three re-infections. Out of the three recrudescence cases, two samples were successfully sequenced and gave interpretable sequences at day 0 and the day of respective recurrence for both *Pfk13* and *Pfcoronin*. As well, the sequencing of the ETF sample was successful for the two molecular markers. For one recrudescence sample, the sequencing was obtained only at day 0 and this was unsuccessful for the day of recurrence.

All sequences had wild-type *Pfk13* allele and wild-type *Pfcoronin* allele for both day 0 and day of recurrence (Table 4).

**Table 4:** Summary of genotyping of parasites and molecular markers analysis.

Drug	Sample ID	Day of recurrence	Parasitemia at day of recurrence	PCR correction	*Pfk13* allele	*Pfcoronin* allele
AL	303	2	4910 (*vs* 1148 at day 0)	NA	WT	WT
AL	344	7	55	negative^a^		
AL	378	21	8730	re-infection		
AL	305	35	72632	recrudescence	WT	WT
AL	306	35	44100	re-infection		
AL	317	35	28700	re-infection		
DP	278	14	2974	recrudescence	WT	WT
DP	260	35	40	recrudescence	WT*	WT*
DP	216	42	495	negative^a^		

*Only sample from day 0 was successfully sequenced.

WT: wild-type

NA: Not applicable.

^a^There were two samples for which microscopy was positive while DNA amplification (PCR correction) was negative, probably due to either false positive microscopy or low-quality DNA. Thus, it was not possible to classify those samples into recrudescence or re-infection.

### Safety outcomes

Generally, drug regimens were well-tolerated. Few minor adverse events were recorded, which did not require a rescue treatment. This included only vomiting in four patients under the ASAQ arm.

## Discussion

More than 10 years after ACT use in Senegal, this study has shown the efficacy and tolerance of these antimalarial drugs in Senegal.

Efficacy and safety of antimalarial drugs has been monitored since the 80 s as highlighted by the finding of chloroquine resistance in this period, yielding to the abandoning of this molecule in 2003^1^. As well, ACT has been evaluated since their first introduction in 2006 in the country and annual TES following WHO protocol is now regularly conducted.

TES has the advantage to prospectively and directly evaluate the clinical and parasitological responses after antimalarial treatment and thus has the potential of early detection of delayed clearance. A change in the national malaria treatment policy should be initiated if the total treatment failure rate is ≥10%, as assessed through TES^16^.

In this study, PCR-corrected ACPR greater than 98% was observed for the three antimalarial combinations drugs. As well, parasitemia cleared rapidly on day-3 in this study.

This demonstrates that ACTs remains highly efficacious, more than ten years after their implementation, consistent with previous studies showing high cure rates and well tolerance of these drugs^17–24^.

Previously, artemether-lumefantrine given as four doses has been associated with a failure rate of 3.6%^25^. However, when this regimen was reviewed by switching to six doses, the high cure rate was restored^26^. This denotes the role of incorrect dosage. In addition, poor patient compliance and poor drug quality can play a major role in causing treatment failure. Thus, in its 2016–2020 Strategic Plan, the Senegal NMCP planned to strengthen coordination in the analytical control of drugs before any marketing authorization is issued, before any distribution of drugs and to monitor drug quality at operational level^27^.

Similarly, the efficacy of ACTs monitored in most African malaria-endemic countries showed overall average efficacy rates of dihydroartemisinin-piperaquine, artesunate-amodiaquine and artemether- lumefantrine of 98.7%, 98.3% and 97.9%, respectively^10,28–35^. Some reports of delayed parasite clearance have been reported in Africa but not consistent over time^10^.

Although treatment failure rate was very low in this study, it is important to analyze those particular samples at the molecular level. In fact, one mechanism of drug resistance emergence is through selection of a resistant clone which may then spread if successful transmission occurs^8^. In this study, *Pfk13* and *Pfcoronin* were analyzed simultaneously. However, in this study, it was not intended to perform a comprehensive analysis of molecular markers of resistance. Instead, previous studies conducted from field isolates in Senegal showed no *Pfk13* mutation associated with artemisinin delayed parasite clearance^36^. *Pfcoronin* is a novel marker that has been discovered using the same approach as that of *Pfk13* but using Senegalese parasites background^15^. In this study, no *Pfcoronin* mutations associated with treatment failure were observed. To date, none of the *Pfcoronin* mutations conferring resistance have been observed in field isolates in Africa^37,38^. Unless slow clearance is observed, as measured by microscopy, the implication of *Pfcoronin* as a genetic basis of artemisinin resistance remains to be elucidated. Further investigations including protein dynamics and intracellular trafficking need to be addressed to better understand the mechanism of artemisinin resistance at the biochemical level^39^. However, consideration should be given in the future to *Pfcoronin* along with *Pfk13* in monitoring molecular markers of artemisinin resistance during efficacy studies.

## Conclusion

This study showed that AL, ASAQ and DP remain efficacious in the treatment of uncomplicated falciparum malaria in Senegal. Continued monitoring of treatment efficacy and for molecular markers of resistance throughout the country is recommended to allow early detection of emerging and spreading resistance and to provide updates for malaria control measures. In addition, it is important that both *Pfk13* and *Pfcoronin* are monitored in sub-Saharan Africa studies.

## Materials and Methods

### Study design

This was a randomized, three-arm, open-label study to evaluate the efficacy and safety of artemether-lumefantrine (AL), artesunate-amodiaquine (ASAQ) and dihydroartemisinin-piperaquine (DP), following a modified WHO 2009 protocol^11^. The study was carried out in two different areas of malaria transmission level (Fig. 1): (1) the health post of Tomboronkoto (Kedougou region), a rural area, located in south where the transmission is high and 2) the health post of Keur Serigne Mbaye Sarr (Diourbel region), an urban area, in the centre where the transmission is moderate. In Tomboronkoto, the malaria incidence rate was estimated at 1049 per 1000 population in 2017. At the same time, in Keur Serigne Mbaye Sarr health post, the incidence rate was 168 per 1000 population^40^.

### Sample size calculation

According to the WHO protocol^11^, a minimum of 73 patients should be recruited to detect a failure rate ≤5%, at confidence level of 95% and an estimate precision of 5%. An additional of 20% was added to take into account lost to follow-up and withdrawals. By applying this guideline, 88 patients were targeted for each drug combination in each site.

### Patients screen and enrolment

Malaria suspected patients were screened using both malaria rapid diagnostic test (RDT) (SD Bioline Pf/HRP2) and microscopy. Patients were enrolled if they were aged 6 months or above, had an axillary temperature ≥37.5 °C at presentation or history of fever during the last 24 hours, a positive *P. falciparum* mono-infection parasitemia of 1,000 to 100,000 asexual forms/μl, ability to swallow oral medication, ability and willingness to comply with the study protocol and visit schedule for the duration of the study and written informed consent from the patient or from the parent or guardian for enrolled children. Infected patients who did not meet inclusion criteria were treated according to the NMCP guideline.

In addition, dried blood spots (DBS) were collected on Whatman filter paper for molecular analysis including parasite genotyping (PCR correction) to differentiate re-infections from recrudescence and to determine molecular markers of artemisinin resistance on samples that were subject to treatment failure.

Key exclusion criteria were any of the following: presence of general danger signs in children aged under 5 years or signs of severe falciparum malaria according to the definitions of WHO^41^, mixed or mono-infection with another *Plasmodium* species detected by microscopy, positive pregnancy test, severe malnutrition, febrile conditions due to diseases other than malaria or other known underlying chronic or severe diseases, regular medication, which may interfere with anti-malarial treatment, history of hypersensitivity reactions or contraindications to any of the medicine(s) being tested or used as alternative treatment(s).

### Treatment and follow-up

Enrolled patients were randomly allocated into the three drug arms: oral administration of AL, ASAQ or DP using the WHO recommended therapeutic dose regimens^11^. The allocation sequence was generated using a randomization table created by an Excel-based application. Patients were observed for 30 min after the drug administration for adverse events or vomiting the study drugs. Any patient who vomited was re-treated with the same dose of medicine and observed for an additional 30 min. If vomiting occurred again, the patient was withdrawn and treated with IV quinine and if necessary, the patient was referred to the nearest hospital. Paracetamol was offered to all feverish patients.

All daily doses were administered at the health facility level under the supervision of the medical staff.

Follow-up visits were scheduled on days 1, 2, 3, 7, 14, 21, and 28 for treatment with AL and ASAQ and additional follow up on days 35 and 42 for DP treatment (the partner drug, piperaquine, has a longer elimination half-life). During the follow-up visit, the patients were subject to clinical assessment and parasitological examination of blood smear to seek for malarial parasites, and DBS was also collected.

### Safety assessment

A physician from the research staff was responsible for monitoring the safety of ACTs. Adverse events were documented through interviews about previous symptoms and about symptoms that have emerged since the previous follow-up visit. A clinical examination was performed to determine any adverse event. The reported events were recorded and any serious adverse event was to be reported to the sponsor.

### Laboratory assessment

#### Microscopic blood examination

Thick and thin smears were performed and slides were examined on days 0 to confirm the meeting criteria and during subsequent scheduled follow-up day or any unscheduled day if the patient returned for being unwell.

Microscopy examination of Giemsa-stained thick and thin blood films was performed to identify parasite species and determine density according to the WHO procedure^42^. Parasite density (per μl) was calculated assuming a white blood cell count of 8000/μl. All slides were read independently by two level 1 malaria microscopists and the average of the two counts was calculated. If any discrepancy was noted (either in species identification or difference of parasite density of >30%), slides were checked by a third independent reader, and parasite densities were calculated by averaging the two most close counts.

### Genotyping of malaria parasites

In order to differentiate a recrudescence (same parasite strain) from a newly acquired infection (different parasite strain), a genotype analysis was performed. DNA was extracted from the DBS on day 0 (before treatment) and during recurrence of parasitemia on day 7 onwards (cases of treatment failure). All DNA samples from patients undergoing parasitemia recurrence were analyzed for genotyping of the highly polymorphic regions *msp1* and *msp2* loci, as recommended by WHO^11^. In addition, a 24 SNP-based molecular barcode using High Resolution Melting qPCR^43,44^ was used. The WHO 2009 protocol requires sequential genotyping of *msp1*, *msp2* and *glurp* genes to discriminate re-infections from recrudescence^11^. The latter is confirmed only when all three markers yield to the same genotype. However, comparable results from *msp1* and *msp2* genotyping and the 24 SNP-based barcodes have been reported. Unlike *msp* genotyping, the SNP-based barcodes present the advantage to be faster and less intensive^45^.

### Molecular markers for antimalarial drug resistance

In the case of confirmed recrudescence as described above, *Pfk13* propeller domain (codon positions: 440–600) and a portion of the *Pfcoronin* gene (codon positions: 31–186) were amplified using a nested PCR assay. Amplicons were sequenced using Sanger method as previously described^13,15^ from extracted DNA on day 0 (before treatment) and during recrudescence of parasitemia on day 7 onwards.

DNA sequences were analyzed using the Geneious Prime software (version 2020.1.2) to identify specific single-nucleotide polymorphism (SNP) related to artemisinin resistance. Sequences generated in this study were submitted into the European Nucleotide Archive (EBI) database under the accession numbers LR782242, LR782250, LR782259, LR782260, LR782261 and LR782262.

### Treatment outcome

Treatment outcomes were classified according to the WHO protocol as early treatment failure (ETF), late clinical failure (LCF), late parasitological failure (LPF) and adequate clinical and parasitological response (ACPR)^11^.

### Data analysis

Data for each study participant were entered into the WHO standardized Microsoft Excel data collection form. This WHO excel sheet was specifically design for the classification of treatment outcome with and without PCR correction^11^.

### Ethical clearance

The study protocol received ethical clearance from the Ethic Committee of the Senegalese Ministry of Health. Written informed consent was obtained from all participants or parents/guardians of the children. The authors have complied with all relevant regulations for work with human participants. This study was registered at the Pan African Clinical Trials Registry on 09 March 2020 under the number PACTR 202003802011316.
